# Time-Dependent Internalization of S100B by Mesenchymal Stem Cells *via* the Pathways of Clathrin- and Lipid Raft-Mediated Endocytosis

**DOI:** 10.3389/fcell.2021.674995

**Published:** 2021-07-26

**Authors:** Ying Zhang, Jing Zhu, Hao Xu, Qin Yi, Liang Yan, Liang Ye, Xinyuan Zhang, Min Xie, Bin Tan

**Affiliations:** ^1^Department of Pediatric Research Institute, Children’s Hospital of Chongqing Medical University, Chongqing, China; ^2^National Clinical Research Center for Child Health and Disorders, Chongqing, China; ^3^Ministry of Education Key Laboratory of Child Development and Disorders, Chongqing, China; ^4^Chongqing Key Laboratory of Pediatrics, Chongqing, China; ^5^Department of Clinical Laboratory, Children’s Hospital of Chongqing Medical University, Chongqing, China

**Keywords:** MSCs, S100B, glioma microenvironment, internalization, clathrin-mediated endocytosis, clathrin, dynamin, lipid rafts

## Abstract

Mesenchymal stem cells (MSCs) are promising tools for cancer therapy, but there is a risk of malignant transformation in their clinical application. Our previous work revealed that the paracrine protein S100B in the glioma microenvironment induces malignant transformation of MSCs and upregulates intracellular S100B, which could affect cell homeostasis by interfering with p53. The purpose of this study was to investigate whether extracellular S100B can be internalized by MSCs and the specific endocytic pathway involved in S100B internalization. By using real-time confocal microscopy and structured illumination microscopy (SIM), we visualized the uptake of fluorescently labeled S100B protein (S100B-Alexa488) and monitored the intracellular trafficking of internalized vesicles. The results showed that S100B-Alexa488 was efficiently internalized into MSCs in a time-dependent manner and transported through endolysosomal pathways. After that, we used chemical inhibitors and RNA interference approaches to investigate possible mechanisms involved in S100B-Alexa488 uptake. The internalization of S100B-Alexa488 was inhibited by pitstop-2 or dyngo-4a treatment or RNA-mediated silencing of clathrin or dynamin, and the lipid raft-mediated endocytosis inhibitors nystatin and MβCD. In conclusion, our findings show that clathrin and lipid rafts contribute to the internalization of S100B-Alexa488, which provides promising interventions for the safe application of MSCs in glioma therapy.

## Introduction

Gliomas are malignant tumors with poor prognosis and a short median survival time and constitute the majority of neoplasms in the central nervous system (Reifenberger et al., 2017). Mesenchymal stem cells (MSCs) are characterized by low immunogenicity and tumor tissue homing and are increasingly used as promising carriers in stem cell-based therapy for gliomas (Smith et al., 2015; Li et al., 2019; Golinelli et al., 2020). However, malignant transformation of MSCs of both human and murine origin has been reported in many studies (Sánchez et al., 2014; Cui et al., 2019; Dai et al., 2020), and a patient with ischemic stroke developed a primitive tumor similar to malignant glioma after receiving stem cell therapy (Berkowitz et al., 2016). All evidence has shown that the safe use of MSCs in clinical applications is facing great challenges.

S100B is a calcium-binding protein with a molecular weight of approximately 12 kDa (Michetti et al., 2019) that is highly expressed in multiple malignant tumors, such as glioma, melanoma, and breast cancer (Wu et al., 2020). S100B can affect tumor development by inactivating p53, the key regulator of tumor inhibition (Wang et al., 2013; Wu et al., 2020). Our previous studies found that exogenous S100B promotes the malignant transformation of MSCs, and this effect may be mediated via its receptor advanced glycation end products (RAGE) (Tan et al., 2018). However, ligand-receptor interactions cannot explain the upregulation of endogenous S100B in MSCs; therefore, it is necessary to reveal the mechanism of endogenous S100B elevation induced by exogenous S100B. A recent study revealed that rat astrocytes take up extracellular S100B by endocytosis in a time-dependent manner (Lasič et al., 2016). Therefore, the possible role of endocytosis in the specific pathway of the S100B protein is worth considering. Endocytosis refers to the process of bringing exogenous proteins, liquid substances, and membrane lipids into the cell through the inward depression of the plasma membrane (Doherty and McMahon, 2009). Endocytic pathways are classified, depending on the coat proteins on the endocytic vesicle, into clathrin-dependent, and clathrin-independent. Clathrin-mediated endocytosis (CME) is a classic and well-characterized endocytic pathway regulated by multiple proteins especially clathrin and dynamin (Mettlen et al., 2018). In the CME process, clathrin-coated pits are initiated by the recruitment of clathrin, and dynamin subsequently acts as a large GTPase to force vesicle scission from the plasma membrane (Thottacherry et al., 2019). Detailed molecular mechanisms of clathrin-independent endocytosis (CIE) pathways remain largely elusive (Sorkina et al., 2018). Still, they can be subdivided depending on the involvement of dynamin (Cendrowski et al., 2016). Lipid rafts are microdomains enriched with cholesterol and sphingolipids, the common feature of many CIE routs is that they often occur at lipid rafts regions (El-Sayed and Harashima, 2013).

In this article, we examined whether and how extracellular S100B is endocytosed by rat MSCs *in vitro*. Here, we report for the first time that extracellular S100B is internalized by MSCs in a time-dependent manner. Furthermore, the internalization of S100B-Alexa488 is restricted by interfering with either the clathrin-mediated or the lipid rafts-mediated pathway. In conclusion, the exogenous S100B in the glioma microenvironment is internalized into MSCs by both CME and lipid raft-mediated endocytosis.

## Materials and Methods

### Cell Culture and Identification

Male Sprague-Dawley (SD) rats aged 3–4 weeks were purchased from the Experimental Animal Center of Chongqing Medical University, China. MSCs were isolated using the adherent culture method as described (Cui et al., 2019). Cells were cultured in Dulbecco’s modified Eagle’s medium (DMEM)/F12 (Gibco; Thermo Fisher Scientific, Waltham, MA, United States) supplemented with 10% fetal bovine serum (FBS; Gibco) in 5% CO_2_ at 37°C and 95% humidity and subcultured when the cell growth confluence was 70–80%. C6 rat glioma cells were cultured under the same conditions as MSCs. To identify the phenotype of MSCs, the expression of MSC surface antigens at passage 4 was detected via flow cytometry. Cells were incubated with phycoerythrin (PE)-conjugated monoclonal anti-rat CD29 (BD Biosciences, Franklin Lakes, NJ, United States), CD45 and CD34 (Abcam, Cambridge, MA, United States), and allophycocyanin (APC)-conjugated CD71 (BioLegend, San Diego, CA, United States), and percp1-conjugated CD90 (BioLegend) antibodies at 37°C for 30 min and subsequently analyzed by a flow cytometer (FACS Canto Plus; BD Biosciences).

### Transfection of Rat MSCs

Mesenchymal stem cells were seeded on 35 mm confocal dishes overnight, and the confluency was 30–50% when the cells were transfected. Small interfering RNAs (siRNAs) were designed and synthesized by Biomics Biotechnologies Inc. (Jiangsu, China) and are listed below.

Cells were transfected with siRNA targeting clathrin heavy chain (si-CHC) or dynamin2 (si-DNM2) or with negative control siRNA (si-NC) using Lipofectamine RNAiMAX (Thermo Fisher Scientific, Waltham, MA, United States) (Table 1). The siRNAs were diluted in Opti-MEM (Thermo Fisher Scientific), added to the cells and mixed gently at room temperature for 20 min, and then serum-free DMEM/F12 (without antibiotic) was added according to the manufacturer’s protocols. Four hours later, the medium was removed and replaced with fresh DMEM/F12 containing 10% FBS. The concentrations of si-CHC and si-DNM2 used in our experiments were 50 and 100 nM, respectively. After 72 h, live cell internalization confocal assays were conducted as described. The knockdown effects were measured with western blot or RT-qPCR experiments as described after 48–72 h of transfection.

**TABLE 1:** Targeted sequence information of specific siRNAs.

**siRNA target**	**Sequence**
CHC	5′-GACUAUGGAGUCAGACAAAdTdT-3′
DNM2	5′-GGGAUGUCCUGGAGAACAAdTdT-3′

### Labeling and Electrophoretic Identification of S100B-Alexa488

Rat recombinant S100B (Cloud-Clone Corp., Wuhan, China) was dissolved in 10 mM phosphate-buffered saline (PBS) to 1 mg/ml, followed by fluorescent labeling with an Alexa Fluor488 Microscale Protein Labeling Kit (Thermo Fisher Scientific) according to the manufacturer’s instructions. To separate the labeled protein from unreacted dye, the conjugate was purified following the steps in the instructions.

Fluorescently labeled S100B (S100B-Alexa488; 500 ng) was subjected to 10% sodium dodecyl sulfate-polyacrylamide gel electrophoresis (SDS-PAGE) under reducing conditions. The gel was fixed in 30% ethyl alcohol (v/v) and 10% ethanoic acid (v/v) for 30 min, washed with ddH2O, and imaged on a Gel Doc^TM^ XR+ (Bio-Rad, Hercules, CA, United States) gel imager. A 532/28 filter and Blue Epi Illumination were used to excite the Alexa Fluor488 dye.

### Capture and Analysis of Confocal and SIM Images

Confocal images were acquired using the A1R laser confocal microscope (Nikon, Tokyo, Japan). A certain number of cells of similar size in each group were randomly selected for capturing. To compare the internalized vesicles and the mean fluorescence intensity (MFI) in each experiment, cells in each group were captured and analyzed under the constant channel parameters (for capturing), diameter and contrast in the same binary layer (for analysis). Pearson’s correlation coefficient and Mander’s overlap coefficient (MOC) was calculated for evaluating colocalization index. Consecutive Z-stack images were captured with the Nikon N-SIM, reconstructed into three-dimensional (3D) images, and processed into dynamic movies. Excitation for the Alexa488, the Alexa 555/TMR/Lysotracker Red, and Dylight649 chromophores or dye was produced by a 488-, 561-, and 638-nm laser, respectively. All images were captured under a 100× oil objective, denoised and analyzed with the NIS-Elements AR software (version 5. 01. 00; Nikon).

### Uptake Assays and Lyso-Tracker Staining

For S100B-Alexa488 and Dextran-TMR uptake assays, when the confluence was 50–60%, cells on each confocal dish were incubated with S100B-Alexa488 (0.1 μM) and 10 kDa Dextran-TMR (0.05 mg/ml, dextran conjugated to tetramethylrhodamine, Thermo Fisher Scientific) simultaneously at 37°C for different time periods (0.5, 1, 2, 6, 12, and 24 h) after 2 h of starvation. For Lyso-Tracker staining, MSCs were incubated with S100B-Alexa488 (0.1 μM) at 37°C for for aforesaid time periods. Then the cells were subjected to fresh medium containing 75 nM Lyso-Tracker Red (Beyotime, Shanghai, China) for 30 min. For endocytic inhibitor assays, the DyLight649-coupled ChromPure Rat Transferrin (15 μg/ml, Jackson ImmunoResearch, West Grove, PA, United States) and the Alexa Fluor 555-coupled cholera toxin subunit B (CTB, 0.5 μg/ml, Thermo Fisher Scientific) are used. MSCs were subjected to S100B-Alexa488 (0.1 μM) and Transferrin-Dylight649 or CTB-Alexa555 for 2 h at 37°C after preincubation with inhibitors as described. For genetic intervention experiments, MSCs were subjected to S100B-Alexa488 (0.1 μM) and Transferrin-Dylight649 (15 μg/ml) for 6 h following 72 h of siRNA transfection. At the end of all internalization assays, cells were gently washed with PBS three times to remove unconjugated dyes. Culture medium without red phenol (Gibco) was added to sustain cell viability, and live cells were subjected to confocal system, images were captured and analyzed with NIS Elements AR software as described above.

### Endocytosis Inhibitor Assays

The endocytosis inhibitors used in our experiments were pitstop-2 (Abcam), dyngo-4a (Selleck, Houston, TX, United States), nystatin (Selleck), and MβCD (Selleck) dissolved in dimethyl sulfoxide (DMSO) or water. MSCs were seeded on confocal dishes overnight, grown to 70% confluency, the preincubation time and concentrations used are pitstop-2 (5 μM) for 30 min, dyngo-4a (20 μM) for 15 min, nystatin (30 μM) for 1 h and MβCD (2 mg/ml) for 1 h, respectively. After pretreatment with inhibitors, cells were subjected to S100B-Alexa488 and Transferrin-Dylight649 or CTB-Alexa555 as described above without washing out the inhibitors. An equal volume of 0.2% DMSO as an inhibitor diluent was used as a control, except for MβCD inhibitor experiments.

### Western Blotting

Total protein was prepared with a Whole Cell Lysate Extraction Kit (KeyGEN, Nanjing, China), a BCA Assay Kit (KeyGEN) was used to detect the concentration, and equal amounts of protein were separated on SDS-PAGE gels. The specific primary antibodies were rabbit anti-rat CHC and DNM2 (1:1000; Bimake, Shanghai, China), S100B (1:1000, Abcam), and mouse anti-rat GAPDH (1:1000; Zhongshan Golden Bridge, Beijing, China). The membranes were incubated with horseradish peroxidase (HRP)-conjugated goat anti-rabbit or goat anti-mouse secondary antibody (Zhongshan Golden Bridge). GAPDH was used as a loading control, the protein bands were detected by ECL (Millipore, Billerica, MA, United States), and the intensities were quantified with Quantity One software (version 4.6.2; Bio-Rad, Hercules, CA, United States).

### Quantitative Real-Time PCR Analysis

Total RNA of cells after 48 h of transfection was extracted with TRIzol reagent (TaKaRa, Dalian, China) and then used for cDNA synthesis using a PrimeScript RT Reagent Kit (TaKaRa). Quantitative real-time PCR was conducted using a SYBR Green Dye Kit (TaKaRa) with gene-specific primers. The primers purchased from TSINGKE Biological Technology (Beijing, China) were as follows: CHC, forward, 5′-GGCGAGTATCAGGCAGCAGTTG-3′, and reverse, 5′-CCCATCTACACAGGCAAAGCAGAC-3′. DNM2, forward, 5′-CGAGGATGGAGCACAAGAGAACAC-3′, and reverse, 5′-GCCACATAGGAGTCCACCAAGTTG-3′. β-actin (forward, 5′-GGAGATTACTGCCCTGGCTCCTA-3′; and reverse, 5′-GACTCATCGTACTCCTGCTTGCTG-3′) was used as the housekeeping gene to normalize mRNA expression. A real-time QuantStudio 3 system (Thermo Fisher Scientific) was used, and the relative gene expression was calculated by the 2^–ΔΔCt^ method.

### Cell Viability Experiments

Mesenchymal stem cells were grown overnight in a 96-well plate. When the cell confluence reached 50–60%, cells in each well were washed three times with serum-free medium, and the medium was replaced with serum-free medium containing different concentrations of pitstop-2 and dyngo-4a. The cells were incubated with drugs for 2.5 h, then CCK8 reagent (ATGene, Taiwan, China) was added to the medium and allowed to react for 4 h at 37°C. A microplate reader (BioTek, Winooski, VT, United States) was used to detect the optical density values at a wavelength of 450 nm.

### Statistical Analysis

Data are presented as the median and interquartile range (IQR) or as the means ± SEM unless stated otherwise by GraphPad Prism 7 software (GraphPad Software Inc., La Jolla, CA, United States). Each experiment was performed at least three times. *P*-values were estimated by Kruskal–Wallis one-way ANOVA with Bonferroni correction or by Mann–Whitney U test unless stated otherwise. Statistical analysis was conducted with IBM SPSS software (version 25; Armonk, NY, United States).

## Results

### Phenotypic Characterization of Rat MSCs

**FIGURE 1:**
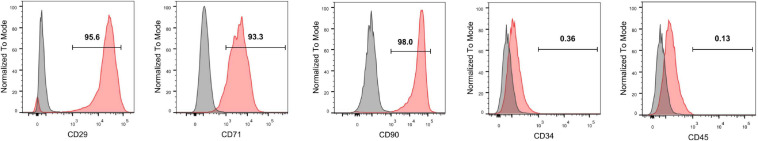
Identification of rat MSCs by flow cytometry. The MSCs were positive for the surface antigens CD73, CD29, and CD90 and negative for CD45 and CD34.

Specific surface markers of rat MSCs at passage 4 were identified with flow cytometric assays. The cells were positive for the MSC surface markers CD29 (95.2%), CD73 (98.3%), and CD90 (98.3%) and negative for the hematopoietic stem cell surface markers CD34 (1.05%) and CD45 (1.56%) (Figure 1).

### Identification of Labeled Recombinant S100B Protein

**FIGURE 2:**
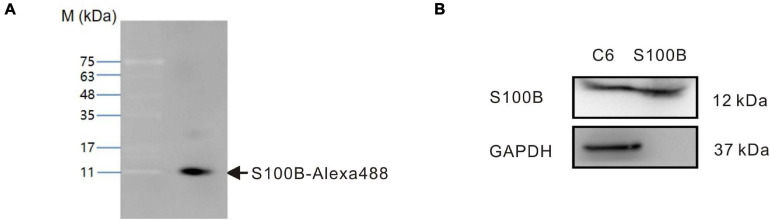
Identification of fluorescently labeled recombinant rat S100B (S100B-Alexa488). **(A)** The S100B-Alexa488 conjugate was analyzed by sodium dodecyl sulfate-polyacrylamide gel electrophoresis (SDS-PAGE) and the gel was imaged using a fluorescence gel imager. The position of the S100B-Alexa488 band is indicated by an arrow. Molecular mass standards are indicated on the left. **(B)** Qualitative analyses of GAPDH and S100B in samples of C6 cell lysates and S100B-Alexa488 conjugate by western blot analysis. S100B was detected in both samples, while GAPDH was only detected in C6 cell lysates.

To enable tracking by fluorescence microscopy, recombinant S100B protein conjugated to AlexaFluor488 dye (S100B-Alexa488) was produced by fluorescently labeling purified recombinant rat S100B. To check the quality of labeling and avoid experimental artifacts caused by unconjugated dye, we performed SDS-PAGE followed by fluorescence imaging. The molecular weight detected upon SDS-PAGE matched the expected molecular weight of S100B (12 kDa), and S100B-Alexa488 was almost completely free of any other impurities (Figure 2A). To test the integrity of surface antigens, S100B-Alexa488 probe and C6 glioma cell lysates (positive control) were subjected to western blot assays. S100B was detected in both samples, while the internal reference GAPDH was only detected in the C6 cell lysates (Figure 2B). These results indicated that recombinant S100B protein was labeled successfully and could be recognized by the S100B antibody.

### Time-Dependent Internalization of S100B-Alexa488 and Dextran-TMR by MSCs

To visualize the possible endocytosis of exogenous S100B, fluorescent dextran (Dextran-TMR), a fluid-phase endocytic marker, was added to the culture medium simultaneously with S100B-Alexa488. Confocal microscopy and structured illumination microscopy (SIM) were used to track this process. To monitor the internalization kinetics of S100B-Alexa488 and Dextran-TMR in detail, we evaluated the number, MFI and colocalization level of fluorescent vesicles internalized per cell at six sequential time points.

**FIGURE 3:**
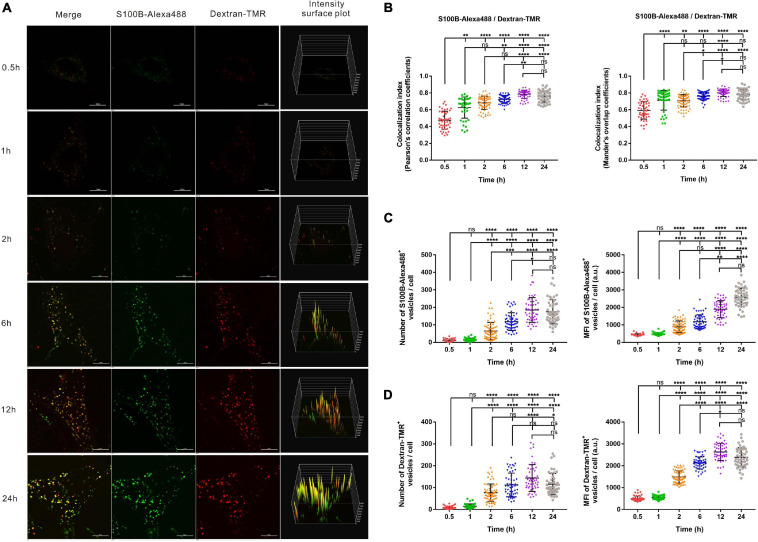
Time-dependent endocytosis of S100B-Alexa488 and Dextran-TMR into MSCs. Cells were incubated with S100B-Alexa488 (0.1 μM) and Dextran-TMR (0.05 mg/ml) simultaneously for the indicated time periods. **(A)** Representative live cell confocal images. Scale bar, 10 μm. **(B–D)** Quantified data (analyzed as described in section “Materials and Methods”) of the indicated time points showing **(B)** the colocalization index (the Pearson’s correlation coefficients and the Mander’s overlap coefficients) of S100B-Alexa488 and Dextran-TMR, the number and mean fluorescence intensity (MFI) of **(C)** S100B-Alexa488- and **(D)** Dextran-TMR-positive vesicles. Data are presented as the means + SD in **(B)** and as the median and interquartile range (IQR) in **C,D**, *n* (0.5 h) = 46 cells; *n* (1 h) = 41 cells; *n* (2 h) = 68 cells; *n* (6 h) = 52 cells; *n* (12 h) = 52 cells; *n* (24 h) = 47 cells. All the experiments were repeated for three times. Asterisks indicate statistical significance between the indicated groups: *P < 0.05, **P < 0.01, ***P < 0.001, ****P < 0.0001, ns, not significant.

The S100B-Alexa488 protein (in green) encountered Dextran-TMR (in red) and thus generated a colocalization signal (in yellow), indicating their strong colocalization (Figure 3A). We evaluated the colocalization level of two fluorescent probes by measuring both the Pearson’s correlation coefficient (PCC) and the MOC, the colocalization index (both the PCC and the MOC) of S100B-Alexa488 and Dextran-TMR was around 0.5 at 0.5 h, this value increased with time and reaches nearly 0.8 at the latter stage, suggesting a partial overlap between the two probes at the beginning of incubation and they have a great potential colocalized in the same organelle in the subsequent endocytic process (Figure 3B). Finally, consecutive Z-stack scanning was performed, and a 3D-reconstructed movie was obtained by using super-resolution SIM (Supplementary Video 1). The 3D movie clearly showed the spatial colocalization of the two fluorescent probes, confirming that they were routed into the same compartments, which is direct evidence that S100B-Alexa488 enters the cell through endocytosis.

Extracellular S100B-Alexa488 were captured in vesicle-like structures, visualized as numerous spots in the cytoplasm, and the MFI represents the content of S100B-Alexa488 in each vesicle. From a short time period perspective, the number and MFI of the two fluorescent probes were hardly detectable at 0.5 or 1 h. Notably, the number of S100B-Alexa488-positive vesicles was clearly detected after 2 h of incubation, and this value doubled at 6 h, peaked at 12 h and slightly decreased at 24 h (Figure 3C, left panel). The MFI of S100B-Alexa488-positive vesicles increased continuously from 0.5 h to a peak value at 24 h (Figure 3C, right panel and Figure 3A, fourth panels). Likewise, the number of Dextran-TMR-positive vesicles increased in a manner similar to that of S100B-Alexa488-positive vesicles (Figure 3D, left panel), and the MFI of it increased over a period of time and slightly decreased at 24 h (Figure 3D, right panel and Figure 3A, fourth panels). The above results showed that the number and the MFI of the two probes had roughly the same variation tendency at different time points, indicating that they have similar time-dependent internalization kinetics.

In general, the above data indicate that exogenous S100B can be efficiently taken up by MSCs simultaneously with the fluid-phase endocytic marker dextran.

### The Transportation and Accumulation of S100B-Alexa488 in Lysosomes

**FIGURE 4:**
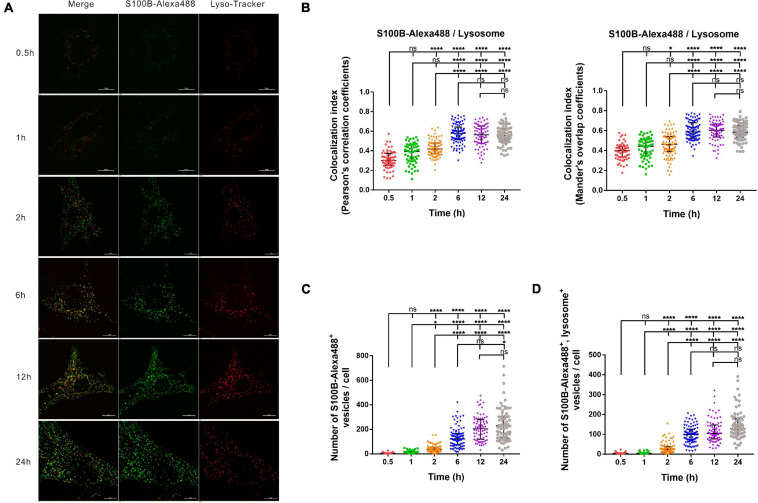
The intracellular transportation and accumulation of S100B-Alexa488 in lysosomes. MSCs were incubated with S100B-Alexa488 (0.1 μM) for the indicated time periods and stained with Lyso-Tracker Red for 30 min. **(A)** Representative live cell confocal images. Scale bar, 10 μm. **(B–D)** Quantified data (analyzed as described in section “Materials and Methods”) of the indicated time points showing **(B)** the colocalization index (the Pearson’s correlation coefficients and the Mander’s overlap coefficients) of S100B-Alexa488 and Lyso-Tracker-labeled lysosomes, **(C)** the number of S100B-Alexa488-positive vesicles, and **(D)** the number of S100B-Alexa488-positive vesicles colocalized with lysosomes. Data are presented as the median and interquartile range (IQR), *n* (0.5 h) = 64 cells; *n* (1 h) = 60 cells; *n* (2 h) = 87 cells; *n* (6 h) = 88 cells; *n* (12 h) = 72 cells; *n* (24 h) = 71 cells. All the experiments were repeated for three times. Asterisks indicate statistical significance between the indicated groups: *P < 0.05, **P < 0.01, ****P < 0.0001, ns, not significant.

To explore the association of S100B-Alexa488 with Lyso-Tracker-labeled lysosomes in the dynamic transportation process, we investigated their colocalization level and the number of fluorescent vesicles per cell using confocal microscopy and SIM. At different time points, S100B-Alexa488 accumulated within the lysosomes (Figure 4A). The colocalization index (both the PCC and the MOC) of S100B-Alexa488 and lysosomes was around 0.3–0.4 at 0.5 h, and this value increased with time, peaked at 6 h and remained stable during the subsequent incubation time, indicating that at the beginning of incubation, a fraction of S100B-Alexa488–positive vesicles reached lysosomes and after prolonged exposure, S100B-Alexa488 was highly colocalized with lysosomes (Figure 4B). In addition, the colocalization of S100B-Alexa488 and lysosomes was reconstructed into a 3D movie that clearly showed the locations of them inside the cell (Supplementary Video 2). Moreover, we observed that during 24 h of incubation, the number of S100B-Alexa488-positive vesicles increased over time (Figure 4C), which was consistent with our previously described results (Figure 3C, left panel), and the number of S100B-Alexa488-positive vesicles that were colocalized with lysosomes increased with time (Figure 4D). The accumulation of S100B-Alexa488 in lysosomes indicated that it is internalized and transported through endolysosomal pathways.

### Internalization of S100B-Alexa488 by MSCs Is Dependent on CME

Clathrin-mediated endocytosis is a well-studied and well-characterized endocytic pathway that is regulated by a host of proteins, including clathrin and dynamin. We investigated whether S100B-Alexa488 is internalized into MSCs by CME by studying the effects of chemical inhibitors and RNA interference (RNAi) of this cellular pathway. To assess the inhibition efficiency, fluorescent transferrin (Transferrin-Dylight649), a well-established protein internalized into the cytoplasm by the CME pathway, was used as a parallel control. DMSO was used to dissolve chemical inhibitors, and we confirmed that DMSO had no adverse effect on endocytosis, as assessed by the number or MFI of S100B-Alexa488 and Transferrin-Dylight649-positive vesicles (Supplementary Figure 1A–C).

**FIGURE 5:**
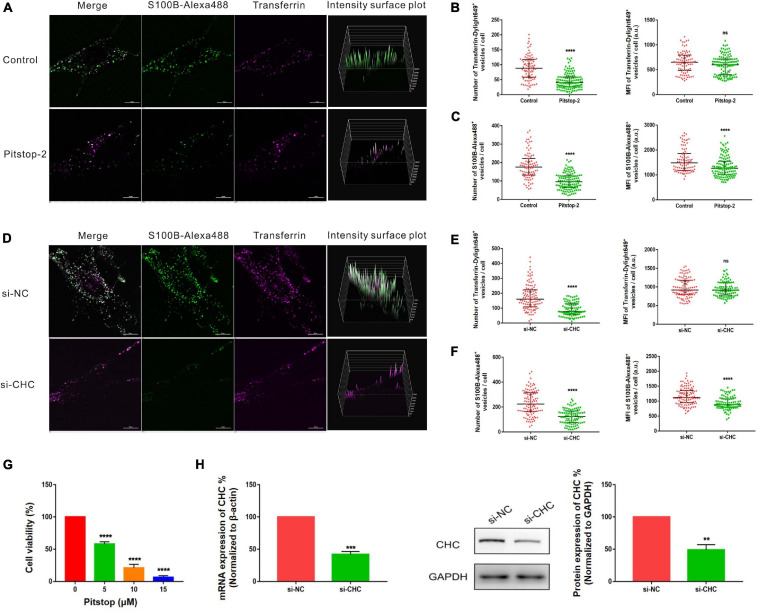
Internalization of S100B-Alexa488 by MSCs is partially dependent on clathrin. **(A–C)** Effects of pitstop-2 on the uptake of S100B-Alexa488 and Transferrin-Dylight649. MSCs were pretreated with pitstop-2 or DMSO and then incubated with S100B-Alexa488 (0.1 μM) and Transferrin-Dylight649 (15 μg/ml) for 2 h. **(A)** Representative live cell confocal images. Scale bar, 10 μm. **(B,C)** Quantified data showing the number and MFI of **(B)** Transferrin-Dylight649- and **(C)** S100B-Alexa488-positive vesicles. **(D–F)** Effects of siRNA-mediated CHC knockdown on the uptake of S100B-Alexa488 and Transferrin-Dylight649. MSCs were transfected with siRNA targeting CHC (si-CHC) or negative control siRNA (si-NC) for 72 h and then incubated with the two fluorescent probes for 6 h as described in section “Materials and Methods”. **(D)** Representative live cell confocal images. Scale bar, 10 μm. **(E,F)** Quantified data showing the number and MFI of **(E)** Transferrin-Dylight649- and **(F)** S100B-Alexa488-positive vesicles. Data are presented as the median and interquartile range (IQR), *n* (control) = 88 cells; *n* (pitstop-2) = 121 cells; *n* (si-NC) = 107 cells; *n* (si-CHC) = 95 cells. All the experiments were repeated for three times. **(G)** Cell viability detected with CCK8 assays after being treated with the indicated concentrations of pitstop-2 for 2.5 h. **(H)** Relative mRNA and protein levels of CHC in cells transfected with si-CHC or si-NC. Data are the means of at least three independent experiments (mean ± SEM). Statistical analysis was done using *t*-tests; **P < 0.01, ***P < 0.001, ****P < 0.0001, ns, not significant versus the control group.

**FIGURE 6:**
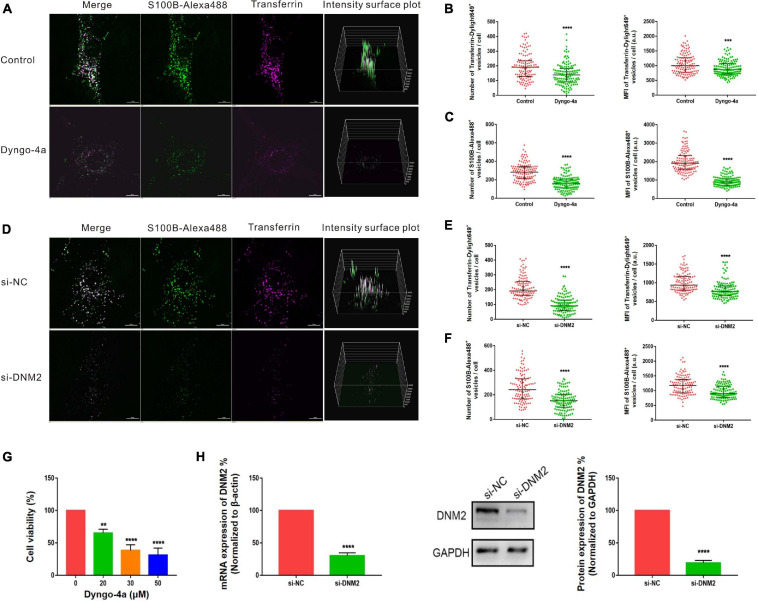
Internalization of S100B-Alexa488 by MSCs is partially dependent on dynamin. **(A–C)** Effects of dyngo-4a on the uptake of S100B-Alexa488 and Transferrin-Dylight649. MSCs were pretreated with dyngo-4a or DMSO and then incubated with S100B-Alexa488 (0.1 μM) and Transferrin-Dylight649 (15 μg/ml) for 2 h. **(A)** Representative live cell confocal images. Scale bar, 10 μm. **(B,C)** Quantified data showing the number and MFI of **(B)** Transferrin-Dylight649- and **(C)** S100B-Alexa488-positive vesicles. **(D–F)** Effects of siRNA-mediated DNM2 knockdown on the uptake of S100B-Alexa488 and Transferrin-Dylight649. MSCs were transfected with siRNA targeting DNM2 (si-DNM2) or negative control siRNA (si-NC) for 72 h and then incubated with the two fluorescent probes for 6 h as described in section “Materials and Methods”. **(D)** Representative live cell confocal images. Scale bar, 10 μm. **(E,F)** Quantified data showing the number and MFI of **(E)** Transferrin-Dylight649- and **(F)** S100B-Alexa488-positive vesicles. Data are presented as the median and interquartile range (IQR), *n* (control) = 123 cells; *n* (Dyngo-4a) = 133 cells; *n* (si-NC) = 112 cells; *n* (si-DNM2) = 116 cells. All the experiments were repeated for three times. **(G)** Cell viability detected with CCK8 assays after being treated with the indicated concentrations of dyngo-4a for 2.5 h. **(H)** Relative mRNA and protein levels of DNM2 in cells transfected with si-DNM2 or si-NC. Data are the means of three independent biological replicates (mean ± SEM). Statistical analysis was done using *t*-tests; **P < 0.01, ***P < 0.001, ****P < 0.0001, ns, not significant versus the control group.

Pitstop-2 acts by blocking the initial binding of ligand to the terminal domain of clathrin, whereas dyngo-4a inhibits dynamin, a membrane scission protein that forms a helical polymer around the constricted neck of a vesicle and mediates its scission from the plasma membrane. To assess the cytotoxicity of these drugs, we used CCK8 assays to screen drug concentrations. The selected concentrations of pitstop-2 and dyngo-4a were 5 and 20 μM, respectively, which corresponded to 50% inhibition of cell viability (Figures 5G, 6G). MSCs were exposed to both S100B-Alexa488 and Transferrin-Dylight649 for 2 h and were pretreated with the drugs or DMSO (control) for the indicated time points. Both fluorescent probes were localized to the periphery and to the perinuclear cytoplasmic regions in the control group, while few fluorescent dots were detected on the plasma membrane in the pitstop-2- or dyngo-4a-treated group (Figures 5A, 6A). There was a significant reduction in the number of Transferrin-Dylight649-positive vesicles in the presence of pitstop-2 or dyngo-4a compared with control cells (Figures 5B, 6B, left panels), confirming that the CME pathway was blocked successfully at the concentrations used. These drugs clearly inhibited the internalization of S100B-Alexa488, as the number and MFI of S100B-Alexa488-positive vesicles were significantly reduced after treatment with pitstop-2 or dyngo-4a (Figures 5C,6C).

Since chemical inhibitors may have many off-target effects, to further verify the above results, siRNA-mediated knockdown was used to investigate the role of clathrin and dynamin in endocytosis. MSCs were transfected with si-CHC and si-DNM2 in the experimental group or si-NC in the control group. The knockdown efficiencies were measured at the mRNA and protein levels, showing that CHC and DNM2 were successfully downregulated (Figures 5H, 6H). The number of Transferrin-Dylight649-positive vesicles was significantly decreased in cells transfected with si-CHC or si-DNM2 compared to those transfected with si-NC (Figures 5E, 6E, left panels), confirming that the CME pathway was inhibited successfully by the specific siRNAs. siRNA-mediated CHC knockdown greatly reduced the number and MFI of S100B-Alexa488-positive vesicles (Figures 5D,F). Likewise, DNM2 silencing significantly reduced the number and MFI of S100B-Alexa488-positive vesicles (Figures 6D,F). The above results suggested that knockdown of CHC and DNM2 inhibits the internalization of S100B-Alexa488.

In summary, these results indicate that inhibition of clathrin and dynamin significantly reduced the uptake of S100B-Alexa488, confirming that S100B-Alexa488 is internalized by MSCs via the CME pathway.

### Internalization of S100B-Alexa488 by MSCs Is Dependent on Lipid Raft-Mediated Endocytosis

**FIGURE 7:**
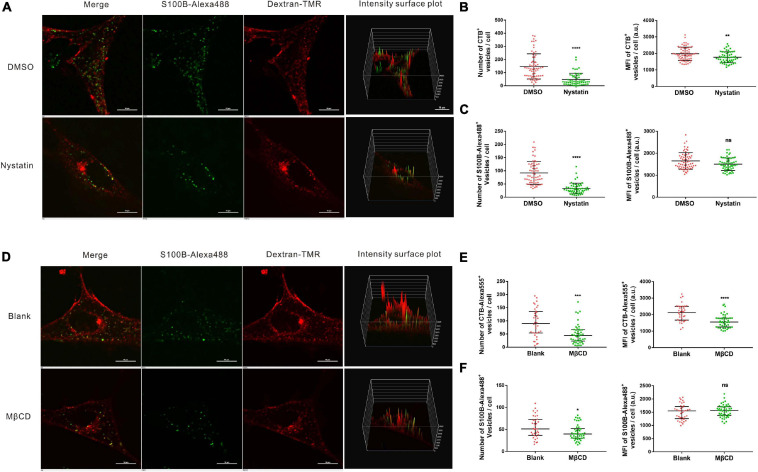
Internalization of S100B-Alexa488 by MSCs is partially dependent on lipid rafts. **(A–C)** Effects of nystatin on the uptake of S100B-Alexa488 and CTB-Alexa555. MSCs were pretreated with nystatin or DMSO and then incubated with S100B-Alexa488 (0.1 μM) and CTB-Alexa555 (0.5 μg/ml) for 2 h. **(A)** Representative live cell confocal images. Scale bar, 10 μm. **(B,C)** Quantified data showing the number and MFI of **(B)** CTB-Alexa555- and **(C)** S100B-Alexa488-positive vesicles. **(D–F)** Effects of MβCD on the uptake of S100B-Alexa488 and CTB-Alexa555. MSCs were pretreated in the absence or presence of MβCD and then incubated with the two fluorescent probes for 2 h as described in section “Materials and Methods”. **(D)** Representative live cell confocal images. Scale bar, 10 μm. **(E,F)** Quantified data showing the number and MFI of **(E)** CTB-Alexa555- and **(F)** S100B-Alexa488-positive vesicles. Data are presented as the median and interquartile range (IQR), *n* (DMSO) = 64 cells; *n* (Nystatin) = 55 cells; *n* (Blank) = 36 cells; *n* (MβCD) = 46 cells. *P < 0.05, **P < 0.01, ***P < 0.001, ****P < 0.0001, ns, not significant versus the control group.

The above results indicated that the internalization of S100B-Alexa488 is dependent on dynamin, which is also involved in multiple CIE pathways. Since the majority of CIE pathways share a dependency on lipid rafts, and membrane cholesterol is an essential component of lipid rafts, we then investigated the role of lipid raft-mediated endocytosis, by using nystatin or MβCD, chemical inhibitors extracting or sequestering cholesterol on the cellular membranes, respectively. To assess the inhibition efficiency, fluorescent CTB (CTB-Alexa555), a maker served to label lipid rafts was used as a parallel control. Treatment with nystatin or MβCD significantly decreased CTB-Alexa555 uptake, assessed by the number and MFI of CTB-Alexa555-positive vesicles, confirming the inhibitory effect of these drugs on lipid rafts (Figures 7B,E). The number of S100B-Alexa488-positive vesicles were greatly reduced in cells treated with nystatin compared with control cells (Figures 7A,C). Likewise, there was a reduction in the number of S100B-Alexa488-positive vesicles after treatment with MβCD (Figures 7D,F). The above results indicate that the presence of cholesterol is indispensable for S100B-Alexa488 uptake, suggesting that lipid raft-mediated endocytosis is involved in the internalization of S100B-Alexa488 by MSCs.

## Discussion

Mesenchymal stem cells have been extensively investigated as tumor-tropic vectors for gene delivery to malignant gliomas (Cao et al., 2018). In the tumor niche, MSCs can be affected by paracrine factors such as cytokines and chemokines (Cui et al., 2014; Tan et al., 2018; Cui et al., 2019). Our previous studies have reported that both GCM and exogenous recombinant S100B protein alone could induce malignant transformation of rat MSCs (Tan et al., 2018). Moreover, the transformation procedure may be mediated through the S100B-RAGE interaction (Tan et al., 2018). However, ligand-receptor interactions cannot explain the upregulation of endogenous S100B in MSCs, which may affect cell homeostasis by inhibiting P53 transcriptional activity (Fernandez-Fernandez et al., 2005). Herein, we report that MSCs take up exogenous recombinant S100B protein through endocytosis. Furthermore, the results indicated that S100B is internalized into the MSC cytoplasm via the pathways of clathrin- and lipid raft-mediated endocytosis.

Previous studies have focused on the intracellular and extracellular signaling pathways of the S100B protein (Donato et al., 2009; Sorci et al., 2013; Chong et al., 2016). However, the possibility of the internalization of S100B, which acts as a secreted protein, has not received much attention. Tracing the internalization of extracellularly introduced fluorescent dextran is a standard method for analyzing fluid-phase endocytosis (Frost et al., 2009; Banerjee et al., 2017). The high colocalization of S100B-Alexa488 with Dextran-TMR confirming the internalization of S100B by MSCs, it was also consistent with the high colocalization of S100B-Alexa488 with lysosomes, as dextran has been reported to be transported into endolysosomes (Humphries et al., 2011; Lasič et al., 2016). The endocytosis of cargo is a dynamic process that includes initiating a budding structure, scission from the plasma membrane, trafficking from endosomes to lysosomes and finally reaching endolysosomes (El-Sayed and Harashima, 2013). Internalized vesicles undergo multiple fusions during the dynamic transportation process (Foret et al., 2012; Scott et al., 2014; Bakker et al., 2017), thus leading to increased MFI of S100B-Alexa488-positive vesicles, indicating its accumulation in intracellular compartments. Moreover, a previous study pointed out that the extracellular cargo reaches the lysosome after the first hours of incubation (Vercauteren et al., 2012), consistent with our results that S100B-Alexa488 is colocalized with Dextran-TMR or lysosomes in the earlier time periods of incubation. In addition, the high colocalization of S100B-Alexa488 with Dextran-TMR or lysosomes lasted for up to 24 h, consistent with previous reports that endosomes containing dextran are localized in lysosomes for a long time (Humphries et al., 2011).

Our findings reveal that S100B and dextran have similar internalization kinetics, probably by a same endocytic pathway. A recent study pointed out that 10 kDa dextran relies on the CME pathway (Li et al., 2015), which is consistent with our findings that the internalization of S100B is dependent on CME. CME is regulated at multiple steps, including initiation, cargo loading, membrane bending, and pit fission, clathrin and dynamin are pivotal regulatory proteins for clathrin coated pit (CCP) formation (Doherty and McMahon, 2009; Mettlen et al., 2018). In this article, chemical inhibitors and genetic approaches were used to inhibit the clathrin and dynamin proteins to verify the regulatory mechanism of S100B internalization. Inhibition of clathrin or dynamin resulted in the blockage of CCP formation, which was confirmed by the reduction in the number of Transferrin-Dylight649-positive vesicles and thus a decrease in the number of S100B-Alexa488-positive vesicles. However, notably, there are some literature reports that pitstop-2 have off-target effects that inhibit CIE (Dutta et al., 2012; Willox et al., 2014). Therefore, it is necessary to consider the possibility of non-specificity of pitstop-2 in CME and its inhibitory effect on CIE.

In addition, our results indicate that dynamin is involved in the internalization of S100B, since dynamin also plays essential roles in multiple CIE pathways (Thottacherry et al., 2019), we focused on the dynamin-dependent CIE pathways, which often take place in lipid raft regions (El-Sayed and Harashima, 2013). We analyzed the role of lipid rafts by assaying the dependence of S100B internalization on cholesterol, and our results indicated that the internalization of S100B was reduced by inhibitors of lipid rafts, suggesting that the dynamin-dependent CIE pathways also participate in S100B uptake. Both the number and MFI of S100B-positive vesicles were reduced after inhibition of clathrin, whereas only the number of S100B-positive vesicles was decreased after inhibition of lipid rafts. In general, our results show that S100B is internalized through CME and in association with lipid rafts. It has been reported that cholesterol depletion by MβCD can not only inhibit lipid raft mediated endocytosis, but also CME (Subtil et al., 1999; Brandenberger et al., 2010). Therefore, the reduction in S100B uptake after MβCD treatment may infer an association with CME. However, the possibility that lipid rafts may be involved in the internalization of S100B should not be discarded and deserves careful consideration. We observed a reduction in S100B uptake in response to nystatin, which provide evidence for the involvement of lipid-raft mediated mechanisms. In addition, Sarnataro et al. (2009), reported that both lipid rafts and clathrin contribute to the efficiency of PrPC protein uptake, and there is evidence that EGFR is capable of endocytosis of both clathrin dependent and non-clathrin mechanisms (Sigismund et al., 2008), which support our findings. Interestingly, none of the inhibitors of either CME or lipid rafts resulted in complete inhibition of S100B-Alexa488 uptake, indicating a contribution from both pathways, further experiments are required to clarify whether inhibition of both pathways had an additive effect on S100B internalization.

The function of internalized S100B protein in MSCs remains to be explored. Although we revealed that CME and lipid rafts contribute to S100B internalization, the inner mechanism of S100B upregulation in MSCs is not clear. Although many studies do not assess the end point of protein internalization, the latest studies have demonstrated that some proteins are not degraded but are instead released from late endosomes; the released proteins maintain biological activity and directly participate in intracellular signaling (Khan et al., 2020). We speculate that this may be one of the possible answers to our earliest question. Furthermore, ligand engagement with receptors continues to signal as they traverse the endocytic pathway (Moore et al., 2018), which means that S100B may affect different intracellular pathways through its receptor RAGE and regulate endogenous S100B expression by a positive feedback mechanism. Therefore, further study is required to determine the outcome of S100B internalization and its applications.

## Conclusion

**FIGURE 8:**
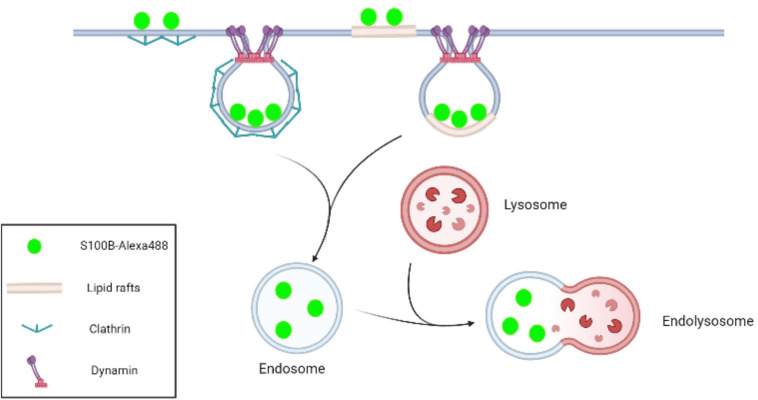
Schematic representation of S100B-Alexa488 internalization by MSCs via the pathways of clathrin- and lipid raft-mediated endocytosis. In the CME process, clathrin-coated pits are initiated by the recruitment of clathrin, and dynamin subsequently acts as a large GTPase to force vesicle scission from the plasma membrane. Lipid rafts are microdomains enriched in cholesterol and sphingolipids. In this work, we observed that S100B-Alexa488 was internalized into the MSC cytoplasm through clathrin- and lipid rafts-mediated endocytosis, and the internalized S100B-Alexa488 may be transported through endolysosomal pathways.

In conclusion, this study confirmed that exogenous S100B protein was internalized into MSCs. Further experiments revealed that S100B endocytosis efficiency was mediated by clathrin, dynamin and lipids rafts. Although a better understanding of the outcome and applications of S100B internalization is still required, this research indicates that paracrine factors in the tumor microenvironment can affect MSCs not only by ligand-receptor interactions but also via intracellular clathrin- and lipid raft-mediated endocytosis (Figure 8).

## Data Availability Statement

The original contributions presented in the study are included in the article/Supplementary Material, further inquiries can be directed to the corresponding author/s.

## Ethics Statement

The animal study was reviewed and approved by the Ethics Committee of the Chongqing Medical University.

## Author Contributions

BT designed and funded the project. YZ performed the experiments and drafted the manuscript. JZ revised the manuscript. HX and QY carried out statistical analyses. LYa contributed to edit the figures. LYe, XZ, and MX contributed to reagent procurement, management, and cell culture. All authors have contributed to the creation of this manuscript for important intellectual content and read and approved the final manuscript.

## Conflict of Interest

The authors declare that the research was conducted in the absence of any commercial or financial relationships that could be construed as a potential conflict of interest.
