# Supplementary material for: Time-Dependent Internalization of S100B by Mesenchymal Stem Cells via the Pathways of Clathrin- and Lipid Raft-Mediated Endocytosis
Source: Front Cell Dev Biol. 2021 Jul 26;9:674995. doi: 10.3389/fcell.2021.674995 (PMC8351554; doi:10.3389/fcell.2021.674995)
Supplement: Supplementary file 1 [file Table_1.docx]

**Supplementary information**

**Figure S1**. DMSO had no adverse effect on the endocytosis of S100B-Alexa^488^ and transferrin-Dylight^649^. MSCs were pretreated with or without 0.2% DMSO for 30min, then the cells were exposed to S100B-Alexa^488^ (0.1µM) and transferrin-Dylight^649^ (15µg/ml) for 2h without washing out DMSO. **(A)** Representative live cell confocal images of MSCs in the presence or absence of DMSO. The green (second panels) and rose red fluorescence (third panels) represent S100B-Alexa488 and Transferrin-Dylight649, respectively, and the white fluorescence shows their colocalization (first panels). The fourth panels show the intensity surface plots of both probes, Scale bar, 10 µm. **(B, C)** Quantified data exhibiting the number and MFI of **(B)** Transferrin-Dylight649- and **(C)** S100B-Alexa488-positive vesicles. ns, not significant compared with the control group.

**Video S1**. Movie of three-dimensional (3D) reconstruction from Z-stack images acquired with N-SIM, showing the spatial co-localization of S100B-Alexa488 (in green fluorescence) and Dextran-TMR (in red fluorescence).

**Video S2**. Movie of 3D reconstruction from Z-stack images acquired with structured illumination microscopy N-SIM, exhibiting the spatial co-localization of S100B-Alexa488 (in green fluorescence) and Lysosomes (in red fluorescence).
