# Supplementary figures and images for: Time-Dependent Internalization of S100B by Mesenchymal Stem Cells via the Pathways of Clathrin- and Lipid Raft-Mediated Endocytosis
Source: Front Cell Dev Biol. 2021 Jul 26;9:674995. doi: 10.3389/fcell.2021.674995 (PMC8351554; doi:10.3389/fcell.2021.674995)

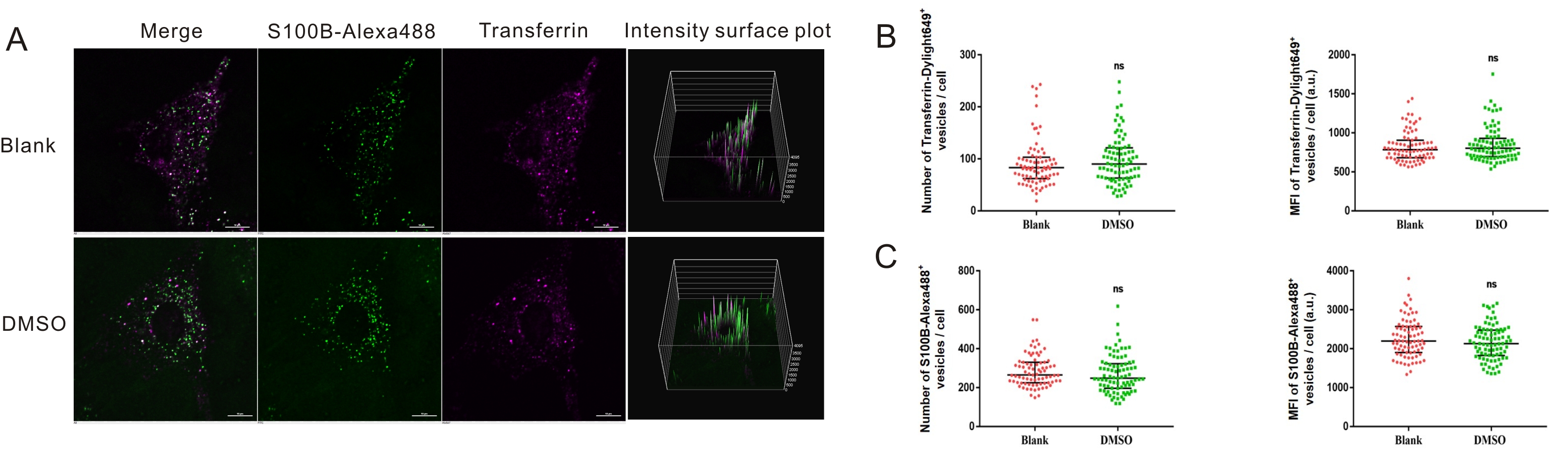

Supplement: Supplementary file 2 [file Image_1.JPEG]
